# The Market Value of Conscience

**Published:** 1888-02-18

**Authors:** 


### The Hospital

A Weekly Institutional Journal of

**Science, Medicine, and Philanthropy.**

*For Hospitals, Asylums, Medical Practitioners, Students, Nurses, Families, Parishes, Congregations, and the Charitable Public.*

Vol. III. No. 73.] [February 18, 1888.

### The Market Value of Conscience.

The world is a battlefield, and in the densest centres of population the fight rages hottest. When men are engaged in hand-to-hand conflict they seldom stop to discuss abstract problems of any kind, least of all those which relate to the rights and wrongs of the question at issue. Public bodies perhaps display less conscience in their business than private individuals. They seek for immediate advantage, prompt and evident results; and so long as they are rewarded with these they do not always too nicely discriminate concerning the nature of the means employed. No one person feels the whole weight of the responsibility attaching to the action of a public body. The head will often place it on the members because they have the voting power; the members will sometimes meanly try to force it on the head because he may have given a biassed lead. In any event, when the actions of a public body are of questionable wisdom, or produce positively injurious results, it seldom happens that any one person takes blame to himself. He soothes his conscience with the reflection that if the whole body had acted rightly instead of wrongly he too would have been on the right side. His intentions were good, but it was of no use trying alone to "swim against the stream."

That line of conduct can never finally dispose of a question. The large world has a wonderful quantity and quality of sense, although it is often very slow in using it. It continues to look steadily and stolidly at objects presented to its view, until sooner or later it is able to say with authority, "That man who looked like a hero is a paper balloon, and only requires the pin-prick of some unlucky circumstance to make him collapse without hope of remedy;" or, "This homely woman who appeared to be of no consequence is made of the truest gold; she purposes always to do only that which gentleness and rightness command, and she does it." Seldom is the world mistaken when it has had sufficient time and knowledge for decision. The verdict of a day or of a year is often reversed; the judgment of a generation or of a century practically never.

It is evident, therefore, that on the very low ground of mere expediency the rights and wrongs of a question demand some consideration; that conscience has a certain value even in the world's not over-scrupulous market. The good or bad quality of a course of conduct cannot always be decided by what may be called its present and apparent merits. Its effects upon other people, and its remote as well as its immediate probable results, must always be taken into full survey. No man or woman would choose to be permanently classed by the world among injurious persons and evildoers. Yet many have been ultimately so classed for the single reason that they possessed feeble and ineffective consciences—consciences which, failing to restrain the unreasoning impulses of disordered feeling, permitted them to enter upon and carry out objects which had no other consequences but those which were entirely injurious.

When unthinking individuals see what foolish and even base things may be said and done among men, without any immediate and evident loss of public esteem and influence, they are apt to think the big, floundering, inchoate world a stupid fool, whose opinions they can afford to despise. That is an entire mistake. There is always here and there one man of intelligence and conscience, one woman of rare moral susceptibility; and the reason of the sterner man and the sensibility of the gentler woman are hurt by base conduct in others. Such persons may appear to be powerless to turn the tide of public opinion at the moment. But the world is in the habit of attaching great weight to the convictions of those whose moral sense and incorruptibility it has tried and proved. Their slow and deliberate verdict is usually held to be a judgment against which there is no appeal. Those who have once come under this condemnation will find great difficulty in resuscitating their reputation for sense and right intention.

It has not been difficult to show, even by the few plain reasons here set forth, that conscience, like articles of commerce, has a definite value in the open markets of the world. But he who strives to preserve the inward monitor alive, and to keep it in robust health on no better grounds than its value in this relation is a poor human creature; poor in intellect, and poor in moral worth. It is curious to note how Englishmen will outwardly approve and applaud the expression of cynical sentiments which sink men to the level of the reasonless and conscienceless animals. A man who did not know better, finding himself in an assembly of educated Britons for the first time, might easily go away with the idea that our fellow-countrymen were hard, sordid, selfish, and unfeeling brutes. It would be a profound and fundamental mistake. The average Briton, of all ranks, has a deep-rooted regard for right and justice; for fairplay in the world's fight; and for that peculiar sentiment well called in holy Scripture, "brotherly kindness." He often does himself an injustice by uttering with his lips what he does not believe in his heart. A pleader is never more sure of winning his cause in this country than when he can, with the boldness of assured conviction, appeal to the deepest sense of duty and right—to the highest conscience of his audience.

In an organization like the hospital world, founded and built up as it is on the noblest sentiments of humanity and the strongest convictions of duty, consience, not only as a marketable property, but still more as an intellectual and moral motive power, occupies a place of peculiar honour. The hospital world does not, and never can, consist merely of patients and officials. It includes in one brotherhood of charity and help all who have the heart to sympathise with suffering, and the will and the power to aid. Those who have charge of hospitals, and particularly they who occupy positions of authority and influence in them, should take every opportunity of developing and maintaining among all their sympathisers and helpers, a high sense of duty and devotion to the great work in which they are engaged. Such a sense of duty is the surest foundation for the permanency of that support on which hospitals so largely depend, and for that efficiency of management which alone can make them worthy of it. An irresponsible, unthinking, and conscienceless behaviour on the part of managers and officials rapidly brings both institutions and their servants into contempt. Nothing disgusts a people more than unworthiness in its priests; and nothing will bring upon benevolent organisations a speedier reprobation than the want of high ideals and worthiness of conduct on the part of those who administer them. No man of sense is ashamed to avow his regard for duty and his belief in its high and eternal sanctions. He only may be ashamed to whom duty is no more than a name, and who has neither the intellect to perceive the foundations on which it rests, nor the nobility of heart to desire its rare and enduring rewards.

